# Allergy clinics in times of the SARS-CoV-2 pandemic: an integrated model

**DOI:** 10.1186/s13601-020-00333-y

**Published:** 2020-06-18

**Authors:** Giacomo Malipiero, Enrico Heffler, Corrado Pelaia, Francesca Puggioni, Francesca Racca, Sebastian Ferri, Lina Spinello, Morena Merigo, Donatella Lamacchia, Giuseppe Cataldo, Melissa Sansonna, Giorgio Walter Canonica, Giovanni Paoletti

**Affiliations:** 1 Personalized Medicine, Asthma and Allergy, Humanitas Clinical and Research Center IRCCS, Via Alessandro Manzoni 56, Rozzano, MI Italy; 2 Department of Biomedical Sciences, Humanitas University, Pieve Emanuele, MI Italy; 3 Department of Medical and Surgical Sciences, University “Magna Grecia” of Catanzaro, Catanzaro, Italy

**Keywords:** Allergy, Asthma, COVID-19, SARS-CoV-2, Pandemic, Immunotherapy, Biologicals, Telemedicine, Digital medicine service, Home delivery

## Abstract

**Background:**

Almost the entire World is experiencing the Coronavirus-Disease-2019 (COVID-19) pandemic, responsible, at the end of May 2020, of more than five million people infected worldwide and about 350,000 deaths. In this context, a deep reorganization of allergy clinics, in order to ensure proper diagnosis and care despite of social distancing measures expose, is needed.

**Main text:**

The reorganization of allergy clinics should include programmed checks for severe and poorly controlled patients, application of digital medicine service for mild-to-moderate disease in well-controlled ones, postponement of non urgent diagnostic work-ups and domiciliation of therapies, whenever possible. As far as therapies, allergen immunotherapy (AIT) should not be stopped and sublingual immunotherapy (SLIT) fits perfectly for this purpose, since a drug home-delivery service can be activated for the entire pandemic duration. Moreover, biologic agents for severe asthma, chronic spontaneous urticaria and atopic dermatitis should be particularly encouraged to achieve best control possible of severe disease in times of COVID-19 and, whenever possible, home-delivery and self-administration should be the preferred choice.

**Conclusion:**

During COVID-19 pandemic, allergists have the responsibility of balancing individual patients’ needs with public health issues, and innovative tools, such as telemedicine and digital medicine services, can be helpful to reduce the risk of viral spreading while delivering up-to-date personalized care.

## Introduction

The recent Coronavirus-Disease-2019 (COVID-19) pandemic has radically changed health priorities and the management of non Covid-19 diseases. At the time of writing (end of May 2020), more than five million people in the world have been infected and about three hundred and fifty thousand have died from COVID 19 [1]. This has resulted in a deep reorganization of health-care systems in order to ensure diagnosis and care for patients and contemporaneously trying to expose them to the minimum risk of contracting the infection in hospital or outpatient clinics. In addition, there is continuous new scientific knowledge about the novel virus that changes the indications on how to treat and prevent it. In this perspective, allergists have to adapt to the change by managing their patients keeping in mind that some allergic diseases of the upper and lower airways, such as allergic rhinitis, chronic rhinosinusitis with nasal polyps (CRSwNP) and asthma have symptoms in common with COVID-19.

## Clinical features of COVID-19 and differential diagnosis with allergic diseases

The susceptibility to and severity of Severe Acute Respiratory Syndrome-Coronavirus-2 (SARS-Cov-2) infection positively correlate with age and comorbidities like hypertension, diabetes and cardiovascular disease [2]. The spectrum of clinical presentations is variable, from asymptomatic and mild clinical symptoms to acute respiratory-distress syndrome (ARDS) [3]. Adults with mild COVID-19 most commonly manifest fever, cough, conjunctivitis, fatigue and anosmia, which in some patients can be accompanied by runny nose and headache. In the more severe clinical presentations, the patient feels worsening dyspnoea and general exhaustion. Additional symptoms such as diarrhea are less common. When the virus affects the lung, it can cause interstitial pneumonia [2]. The most severe manifestations appear to be related to an excessive immune system response (“cytokine storm”) that leads to development of ARDS and respiratory failure requiring invasive ventilation.

**Fig. 1:**
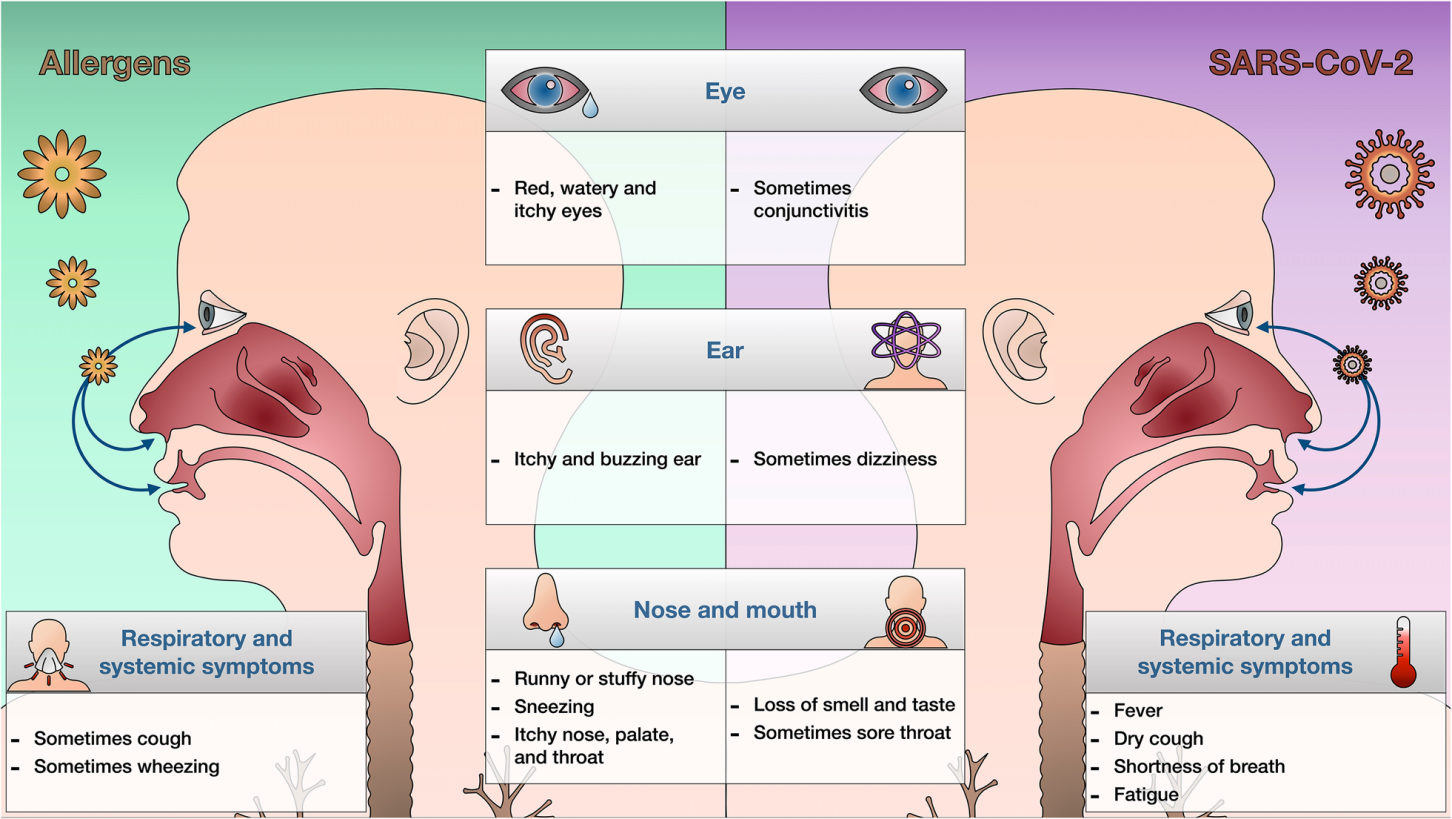
Differential diagnosis of allergic airway diseases and SARS-CoV-2 infection

Some allergic diseases, like allergic rhinitis, CRSwNP and asthma, can simulate symptoms of COVID-19: cough and dyspnoea are shared with asthma, while runny nose and headache are frequent symptoms in allergic rhinitis and CRSwNP. In addition, this pandemic started in the spring season when the majority of patients with seasonal allergies suffers from partially similar symptoms. Anxiety is associated to both chronic airways diseases and COVID-19 pandemic, acting as a confounding factor that should be carefully factored in when interpreting subjective symptoms. From a different perspective, it has been reported that COVID-19 patients might present with skin signs and symptoms of urticaria and eczema resembling those acute urticaria or drug reactions, thus posing a diagnostic challenge to dermatologists and allergists [4]. Therefore, in this kind of patients, it is important to focus attention on symptoms typical for COVID-19, manly fever but also excessive fatigue and impaired sense of smell or taste, to obtain a correct diagnosis (Fig. 1). Two apps have been developed to assess both COVID-19 (MASK-COVID) and asthma/rhinitis (MASK-air, available in 18 languages). A combined MASK-air-COVID app will be launched and will differentiate rhinitis/asthma symptoms from those of COVID-19 (personal communication form Prof. Bousquet).

## The need for mitigation efforts

Healthcare systems have been implemented to ensure citizens’ right of receiving appropriate and prompt care, as declared by the bill of right of many European Countries. In pandemics, unanticipated stresses challenge healthcare systems efficiency and performance, given the acute extraordinary shifts in patients’ needs and allocation of resources. Of course, a number of measures can be enacted to slow the pace of epidemic spread and keep health-care systems accessible to everyone. That would translate into a more favorable distribution of resources, meantime allowing for serological tests, results of randomized double-blind placebo-controlled trials and mass vaccination to become available.

The first and obvious measure is quarantining of infected individuals, which is nonetheless limited by the challenge of identifying and isolating asymptomatic individuals and the high false negative rate of microbiological screening tests [5]. Next, use of protective equipment, especially by health-care providers, is a mainstay of mitigation strategy but might be limited by shortage of material or misuse due to lack of operative skills. In the context of uncontrollable spreading of communicable diseases, social limitations and home confinement are extreme measures, even though made more bearable by the availability of social digital technologies. To make social distancing more feasible, a number of issues should be addressed and, in the context of care delivering, reducing unnecessary direct patients’ interaction with health-care providers is a priority requiring maximum efforts to adapt to and may be prolonged to the post-pandemic period, according to transmission dynamics of SARS-CoV-2 [6]. There is particular concern for aging individuals, where coexisting comorbidities and consequent polypharmacy carry the risk of adverse events in terms of loss of control of chronic diseases, lack of therapeutic adherence, drug-related adverse events and need for urgent care, which would put both the patient and health care providers at risk of infection. In addition, uncontrolled chronic airway diseases could contribute to SARS-CoV-2 dissemination through coughing or sneezing, especially during periods of increased pollen exposure. Delivering targeted educational programs and action plans to enact in case of disease exacerbation is essential. Patients should be routinely checked about their technical skills (e.g. inhaler use) and treatment adherence and remotely monitored for initial signs of physical or psychological decompensation, possibly by using mobile health technology [7].

## Allergy clinic at COVID-19 times: the dawn of a new era for telemedicine and digital medicine

Since allergic diseases are highly heterogeneous in severity and risk of exacerbation, both at inter- and intra-patient levels, in the event of acute outpatients’ service rationing aimed at mitigating virus spread, patients’ access to allergy services has to be rationalized [8, 9]. A gradual escalation in acute service reduction would be preferable but might not be applicable in case of tumultuous spread of the virus. Similarly, “phase 2” re-opening policies should be based on gradualism and prudence. To this aim, a risk-stratified approach can be applied by considering patients’ clinical needs and in accordance to local and institutional policies. Therefore, acute service reduction can be arranged through combinations of measures that could change form nation to nation according to local epidemiology and socio-political trends. In such a highly dynamic setting, it is difficult to forecast how long the pandemic will last and whether subsequent “waves” of COVID-19 will require additional lockdowns before herd immunity is reached or a vaccine becomes available. Thus integrated planning is essential to keep allergy clinics and allergists operative in the long-term.

In general, acute access to allergic emergency or life-threatening acute events is mandatory even during pandemics. As to outpatients, scheduled in-person visits for severe and poorly controlled allergic diseases should be regularly provided, by instructing patients to enter the clinic in a precise time-range and respect social distancing. To reduce the risk of in-hospital spread of the infection, COVID-19 and COVID-19 free-zones can be created by predisposing check-points at COVID-19 free-zone entrance to detect patients with fever (> 37.5 °C), screen for symptoms of active infection and/or contact with COVID-19 positive individuals. Conversely, accesses for mild-to-moderate disease in well-controlled patients can be transitioned to a digital medicine service (DMS), including phone, video and email consults [10]. Telehealth has the advantage of respecting social distancing, thus reducing the risk of viral transmission. When patients experience worsening symptoms requiring timely evaluation, digital tele-triaging should be arranged in order to segregate patients suitable for remote monitoring and therapeutic adjustment (for example, by providing them with an up-to-date action plan) from those requiring in-person visits and access to health-care facilities. Psychological and physical barriers to treatment adherence and comorbidities causing loss of control might be safely addressed and resolved by teleconsultations. Moreover, digital teletriaging of patients experiencing worsening respiratory symptoms can provide an invaluable aid at suspecting COVID-19 and activating general practitioners to monitor and/or investigate suspected patients [11]. Patients waiting for diagnostic work-up or experiencing troublesome lack of comfort with therapeutic recommendations can access to digital platforms in order to contact their caring specialist. Objective questionnaires administered on-line may help deal with patients who poorly perceive their symptoms. We specify that some of these clinical interventions and ascertainments may be provided by health providers collaborating with allergists in multidisciplinary teams, such as specialized nurses, dietitians, ENT physicians and psychologists.

It should be noted that virtual care has to be delivered according to national laws and by licensed providers. This could represent a problem where laws governing virtual care are not available or not up-to-date to ever modernizing tools. Standardized digital platforms should be provided to patients and health-care providers, accessible through a digital identity, in order to avoid misconducts and retain information in case of legal allegations [12]. Meantime, other more common and widespread tools might be used, provided a properly informed consent to being treated via telemedicine has been obtained from the patient, when admitted by national laws on general data protection.

Diagnostic work-up can be postponed in the great majority of allergic diseases, provided affected patients are well instructed about avoidance of potential environmental triggers and treatment of acute events, also based on written action plans [13, 14]. Anyway, diagnostic procedures should be reconsidered as soon as pandemic becomes more controlled and confinement de-escalation begins.

Therapeutic management deserves particular attention. Whenever possible, therapeutic maneuvers not requiring hospital access should be domiciled.

In SARS-Cov-2 negative, low-risk individuals, allergen immunotherapy (AIT) should not be stopped and actually can be started in naive patients provided the required infrastructure is in place during the pandemic [15]. We underline that sublingual immunotherapy (SLIT) for airborne allergens allows not suspending AIT and can be a valid and safe alternative for patients about to undergo SCIT, as far as a drug home-delivering service is activated by the local Pharmacy [9]. This can prove a fruitful advantage of SLIT over subcutaneous immunotherapy (SCIT), in keeping patients adherent to AIT; transition from SCIT to SLIT might be considered as a possible management strategy during COVID-19 pandemic. SCIT for hymenoptera venom allergy should be continued although increasing administration interval can be considered during long-term maintenance. In confirmed or suspected COVID-19 patients, AIT should not be started and should be temporarily interrupted if already prescribed. AIT-specific recommendations are summarized in Table 1 [15].

**Table 1:** AIT recommendations (adapted from Klimek et al. 2020), [19]

Recommendations in non COVID-19 individuals
Interrupting subcutaneous immunotherapy is not advised. Especially in potentially life-threatening allergies, such as venom allergy, SCIT should be regularly continued. The possibility of expanding injection intervals in the continuation phase should be checked and may be beneficial
Interrupting sublingual immunotherapy is not advised. Supply the patient with sufficient medication for a minimum of 14 days isolation
Sublingual immunotherapy can be taken at home. The intake of SLIT by the patient at home or any place is advantageous in avoiding contact with potentially infected persons
Both subcutaneous and sublingual immunotherapy can be continued in the current COVID-19 pandemics, in any asymptomatic patient without suspicion for SARS-CoV-2 infection and/or contact with SARS-CoV-2 positive individuals, in any patient with negative test result (RT-PCR) or in any patient after an adequate quarantine or with detection of serum IgG to SARS-CoV-2 without virus-specific IgM
Preparedness of your Allergy clinic is imperative to cope with COVID-19. Follow World Health Organization (WHO) guidelines and advice staff accordingly
These recommendations are conditional since there is paucity of data and they should be revised regularly with incoming new information on COVID-19

Recommendations in COVID-19 diagnosed cases or suspicion for SARS-CoV-2 infection
Interrupting subcutaneous immunotherapy is advised
Interrupting sublingual immunotherapy is advised
Both subcutaneous and sublingual immunotherapy should be discontinued in symptomatic patients with exposure or contact with SARS-CoV-2 positive individuals, or patients with positive test results (RT-PCR)

First line anti-allergy agents, such as anti-histamines and low-dose steroids, are not likely to affect immunocompetence and should not be discontinued during the pandemic (for disease specific recommendations, see “Disease-specific management” section), provided systemic corticosteroid posology remains under the immunosuppressive threshold [16–18] and that all strategies to reduce systemic corticosteroid use should be applied independently from the pandemic [19]. Notably, regular steroid-based regimens, including inhaled and oral corticosteroids, should not be stopped, since abrupt interruption would bring along an increased risk of adrenal insufficiency in case of acute stressing events, as COVID-19 is [20]. Conversely, a pulsed dose of steroids could result in a protective effect in such a circumstance [21].

Second-line steroid sparing immunomodulating agents are potentially detrimental on immunity although clear-cut distinctions exist between nonspecific immunosuppressive drugs, like cyclosporine and azathioprine, and biological agents targeting discrete molecular pathways associated with type 2 allergic inflammation. In fact, besides inducing nonspecific suppression of anti-infective immunity, nonbiological agents are strong inducers of hepatic cytochromes, thus requiring plasma level monitoring when used in combination with antimicrobial agents [22]. According to a WAO paper [23], no definitive evidence is presently available to obligate clinical decision making. As anti-IgE, ant-IL5/IL5R and anti IL4/IL3R biologics are not likely to induce immunosuppression or interfere with virus clearance (actually the contrary seems to hold true, at least for omalizumab [24] and dupilumab [25], while mepolizumab treatment was not associated with worse immunological response to viruses [26, 27]), they can be safely continued. Anyway, until evidence-based data are available, some countries, like the UK, have advised home confinement for at least 12 weeks to patients with severe asthma on systemic immunosuppressants or biological therapy. Steroid-sparing effect of biologics is a potential advantage over steroid therapy since patients on chronic steroid therapy can be affected by subclinical adrenal insufficiency [20]. Depending on the specific monoclonal antibody, in-site administration or home-delivery and self-administration can be the preferred choice. When patients live far away from the Centre, National Networks can be activated in order to identify a local near-home Centre for in-site administration (in Italy the Severe Asthma Network in Italy (SANI) [28, 29]). In case of infection, discontinuation should be discussed on a case-by-case basis.

We should also consider the complexity of allergic patients and the synchronous atopic involvement of multiple organs that interface with the environment’. Therefore, as far as precision medicines targeting nodal pathogenic pathways allow to treat “atopy” as a whole, we claim priority and lower prescription threshold could be assigned to biologicals, given the good safety profile, the lack of interference with drug metabolism and the favorable effect on treatment adherence. The regulatory agencies should address these new needs in pandemic times and coordinate with health care purchasers to make biological more accessible.

Clinical research has been seriously threatened by COVID-19 epidemics. New recruitments in ongoing clinical trials have been temporarily suspended. Investigators can consult with sponsors in order to reassess follow-up visit schedules and amend protocol requirements to facilitate online visits. When accorded with the Sponsor and local Pharmacy, investigational product home-delivery service and a digital medicine service can be activated to administer scheduled questionnaires and monitor domiciliary drug self-administration [9].

**Fig. 2:**
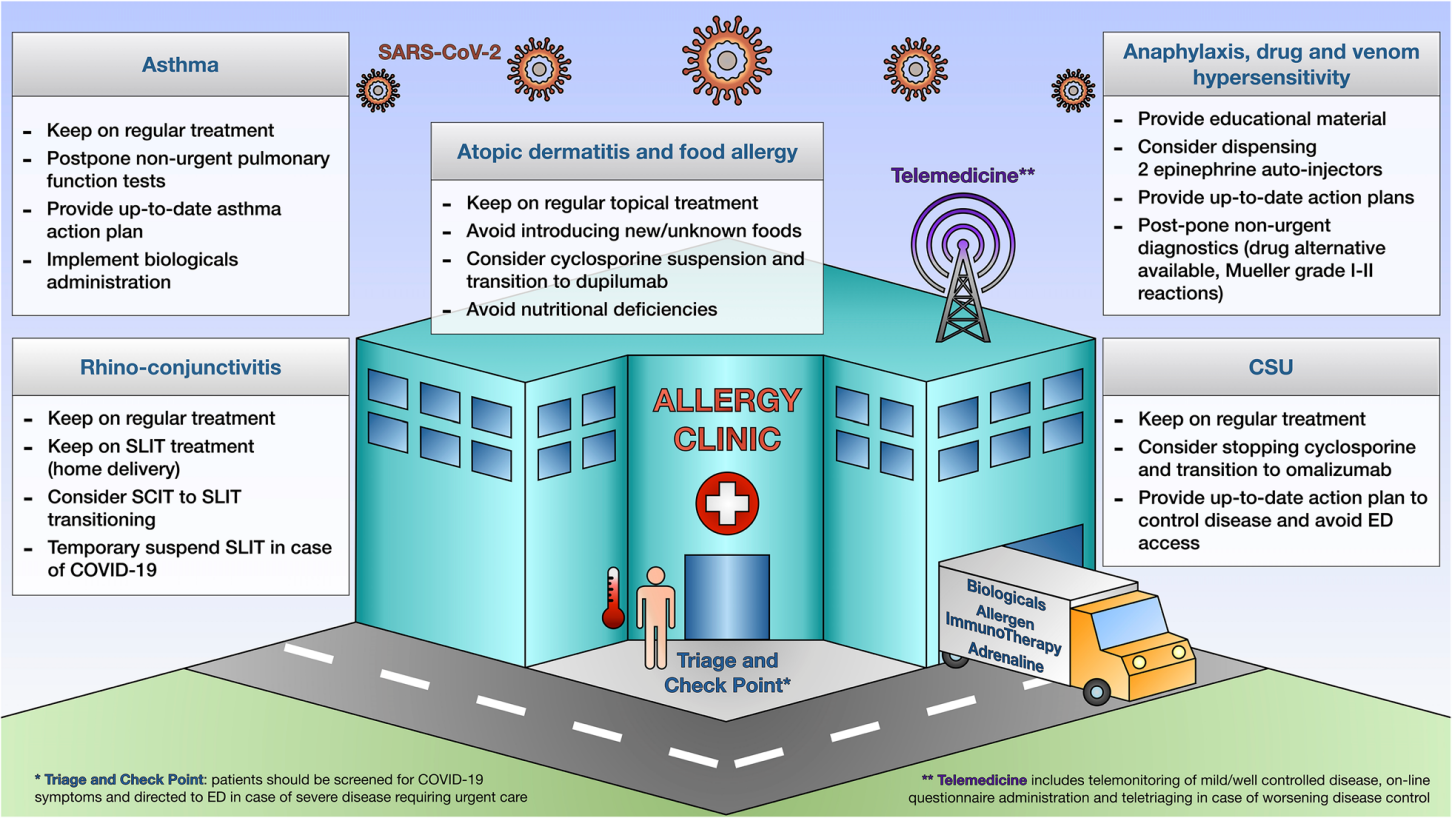
An integrated model of tele- and digital-allergy clinic during COVID-19 pandemic. Suggested actions to be implemented in order to re-organize an allergy clinic are reported in boxes for asthma, rhino-conjunctivitis, atopic dermatitis and food allergy, anaphylaxis, drug and venom hypersensitivity and CSU. The truck represents the home-delivery service that should be activated for biologicals, AIT and self-injectable adrenaline

Disease-specific measures and therapeutic recommendations are rapidly provided in the following sections and graphically resumed in Fig. 2. In the case of atopic multimorbidity, priority should be given to uncontrolled/severe components over well-controlled/mild ones.

## Disease-specific management

### Asthma

Asthma-specific recommendations from Global Initiative for Asthma (GINA) are reported in Table 2 [30]. Data are controversial regarding the interaction between asthma and COVID-19 as European and Chinese data suggest a lower risk for asthmatic patients to contract the infection [31] while U.S. data suggest the opposite [32]. Probably this association is modulated by the severity of asthma, age class, and levels of indoor and outdoor, allergen and pollution exposures. With knowledge evolving, GINA and the British National Institute for Health and Care Excellence (NICE) guidelines recommend not stopping the regular treatment regimen, including inhaled and oral corticosteroids [30, 33], since abrupt interruption would bring along an increased risk of acute/severe exacerbations or adrenal insufficiency [21]. Every patient should be provided with a detailed and convenient action plan to deal with potential loss of control [13], and invited to regularly use mobile health technology apps to self-monitor symptoms of their asthma [7]. Aerosol generating procedures are discouraged unless essential and lung function testing should be postponed whenever possible [30].

**Table 2:** Asthma-specific recommendations from Global Initiative for Asthma (GINA) as reported on GINA website [34]

People with asthma should continue all of their inhaled medication, including inhaled corticosteroids, as prescribed by their doctor
In acute asthma attacks patients should take a short course of oral corticosteroids if instructed in their asthma action plan or by their healthcare provider, to prevent serious consequences
In rare cases, patients with severe asthma might require long-term treatment with oral corticosteroids (OCS) on top of their inhaled medication(s). This treatment should be continued at the lowest possible dose in these patients at risk of severe attacks/exacerbations. Biologic therapies should be used in severe asthma patients who qualify for them, in order to limit the need for OCS as much as possible
Nebulizers should, where possible, be avoided for acute attacks due to the increased risk of disseminating COVID-19 (to other patients AND to physicians, nurses and other personnel) Pressurized metered dose inhaler (pMDI) via a spacer is the preferred treatment during severe attacks. (Spacers must not be shared at home) While a patient is being treated for a severe attack, their maintenance inhaled asthma treatment should be continued (at home AND in the hospital)
Patients with allergic rhinitis should continue to take their nasal corticosteroids, as prescribed by their clinician
Routine spirometry testing should be suspended to reduce the risk of viral transmission, and if absolutely necessary, adequate infection control measures should be taken

### Allergic rhino-conjunctivitis

Allergic rhino-conjunctivitis-specific recommendations from allergic rhinitis and its impact on asthma (ARIA) and European Academy of Allergy and Clinical Immunology (EAACI) are reported in Table 3 [15, 34]. Allergic rhinitis visits should not be prioritized and can be safely postponed unless exceptional circumstances supervene. Intranasal steroids do not induce immunosuppression and are not to discontinue [34]. As stated above, optimal disease control is mandatory as sneezing in asymptomatically infected patients can contribute to viral spread; mobile applications can contribute in improving optimal self-management of rhinitis [7]. For AIT-specific recommendations, see Table 1 [15].

**Table 3:** Allergic rhino-conjunctivitis-specific recommendations from ARIA/EAACI (from: Bousquet et al. 2020) [37]

With the current knowledge, in patients with COVID-19 infection, intra-nasal corticosteroid (including spray) can be continued in allergic rhinitis at the recommended dose
Stopping local intra-nasal corticosteroid is not advised. Suppression of the immune system has not been proven and more sneezing after stopping means more spreading of the Coronavirus
These recommendations are conditional since there is a paucity of data and they should be revised regularly with new knowledge

### Chronic rhinosinusitis

We are unaware of specific recommendations from medical societies about this topic. For reasons analogous to those specified for allergic rhino-conjunctivitis, it is commonsense to keep patients on regular treatments. Patients with uncontrolled disease or waiting for Ear-Nose-Throat (ENT) surgery could be transitioned to biological treatment whenever possible.

### Atopic dermatitis and food allergy

Disseminated skin viral infections are a major complication of severe/uncontrolled atopic dermatitis. It is unknown whether increased risk of SARS-Cov-2 infection or disease severity is associated with atopic dermatitis due to impaired cutaneous barrier. Patients on immunosuppressive therapy for severe atopic dermatitis should be strictly monitored or transitioned to safer anti-IL4/IL13 immune-modulatory agents, as advocated by the European Task Force on atopic dermatitis [35, 36]. Safe use of Dupilumab in severe AD patients affected by COVID-19 has been recently reported [37]. Food allergy and allergic gastrointestinal diseases are prevalent comorbidities in atopic dermatitis, especially in children; careful evaluation is needed and food challenge should be performed to prevent unnecessary food avoidance interfering with growth and micronutrients balance [38]. Food allergy in asthma is of particular concern since it has been associated with fatal asthma. Asthmatic patients with food allergies may need to be provided with epinephrine auto injectors (see the following section on anaphylaxis management) and should avoid introducing new foods in their diets during the pandemic as long as the risk of contagion is high [38].

### Anaphylaxis

A modified anaphylaxis management algorithm during COVID-19 pandemic has been proposed by Casale et al. [39]. Patients are invited to carry two epinephrine injectors and to activate emergency services only when a second administration of epinephrine fails to control symptoms. It might be prudent to proactively discuss the modified management of anaphylaxis, if feasible, e.g., via telemedicine. Home delivery service of self-injectable adrenaline should be implemented, provided the patient has been instructed on the underlying disease, triggers avoidance measures and proper use.

### Drug allergy, hymenoptera venom allergy and urticaria/angioedema

Expert recommendations and consensus on these topics during COVID-19 epidemics are missing at the time of this manuscript being written. As a general principle, we maintain that ongoing treatment for these conditions should not to be discontinued in order to keep disease under control as far as these treatments are not likely to increase the risk of SARS-Cov-2 infection or complicate COVID-19 course in case of infection. A possible exception might be represented by patients on immunosuppressive regimens (e.g. cyclosporine for chronic spontaneous urticaria), in whom shift to a biological agent with a more favorable safety profile might be considered on a case-by-case basis (e.g. close contact with infected individuals, active infection) [40]. For new evaluations, diagnostic procedures should be postponed during the epidemics peak and reconsidered on a second time, unless urgently needed (e.g. severe reactions to Hymenoptera venom). We highlight that urticaria can be a presenting sign of COVID-19 and allergy to drugs used to treat COVID-19 can present with rashes that may involve the allergist’s evaluation [4].

## Conclusion

We are now in the middle of the storm and we do not know how long it will last or whether additional consequences are on the horizon. In these times of suspense, we have the responsibility of balancing individual patients’ needs with public health issues. To our mind, a black-and-white thinking does not suit a scientific way of addressing theoretical and practical problems. We therefore advocate for a risk-stratified approach aimed at conjugating COVID-19 and non-COVID-19 health care needs of allergic patients. We have to reshape our practice and optimize those innovative tools that help us reduce the risk of viral spreading while delivering up-to-date personalized care. A new era of integrated precision-digital medicine is knocking at the door.

## Data Availability

Not applicable.

## Abbreviations

ACE-2: Angiotensin-converting enzyme-2
AIT: Allergen immunotherapy
ARDS: Acute respiratory distress syndrome
ARIA: Allergic rhinitis and its impact on asthma
COVID-19: Coronavirus-Disease 2019
CRSwNP: Chronic rhinosinusitis with nasal polyps
DMS: Digital medicine service
EAACI: European Academy of Allergy and Clinical Immunology
ENT: Ear-Nose-Throat
GINA: Global initiative for asthma
NICE: National Institute for Health and Care Excellence
OCS: Oral corticosteroids
pMDI: Pressurized metered dose inhaler
SANI: Severe Asthma Network in Italy
SARS-CoV-2: Severe Acute Respiratory Syndrome-Coronavirus-2
SCIT: Subcutaneous immunotherapy
SLIT: Sublingual immunotherapy
TLR: Toll-like receptor
WHO: World Health Organization
